# Antibiotic Selection and Outcomes in Patients With Monomicrobial and Polymicrobial *Vibrio* Necrotizing Fasciitis

**DOI:** 10.1155/cjid/9414923

**Published:** 2026-09-26

**Authors:** Jiun-Liang Chen, Chun-Hao Chen, Hung-Yen Chen, Cheng-Ting Hsiao, Shu-Fang Kuo, Chien-Hui Hung, Hsing-Jung Li, Tsung-Yu Huang, Yao-Hung Tsai

**Affiliations:** ^1^ Department of Orthopaedic Surgery, Chia-Yi Chang Gung Memorial Hospital, No. 6 West Sec Chia-Pu Road Putz City, Cha-I 613, Taiwan; ^2^ College of Medicine, Chang Gung University, Taoyuan 33303, Taiwan, cgu.edu.tw; ^3^ Department of Emergency Medicine, Chiayi Chang Gung Memorial Hospital, Chiayi 61363, Taiwan, cgmh.org.tw; ^4^ Microbiology Treatment and Research Center, Chiayi Chang Gung Memorial Hospital, Chiayi 61363, Taiwan, cgmh.org.tw; ^5^ Department of Laboratory Medicine, Chiayi Chang Gung Memorial Hospital, Chiayi 61363, Taiwan, cgmh.org.tw; ^6^ Department of Medical Biotechnology and Laboratory Sciences, College of Medicine, Chang Gung University, Taoyuan 33303, Taiwan, cgu.edu.tw; ^7^ Graduate Institute of Clinical Medical Sciences, College of Medicine, Chang‐Gung University, Taoyuan 33302, Taiwan, medcol.mw; ^8^ Department of Internal Medicine, Division of Infectious Diseases, Chiayi Chang Gung Memorial Hospital, Chiayi 61363, Taiwan, cgmh.org.tw; ^9^ Department of Pediatrics, St. Martin De Porres Hospital, Chiayi 60069, Taiwan, stmartinhospital.com

**Keywords:** empirical antibiotic, necrotizing fasciitis, polymicrobial, third-generation cephalosporins, *Vibrio*

## Abstract

**Background:**

Necrotizing fasciitis (NF) caused by *Vibrio* species is a rapidly progressing, life‐threatening infection. The purposes of this study were to characterize polymicrobial *Vibrio* NF and compare its clinical features, microbiology, antimicrobial therapy, and outcomes with those of monomicrobial *Vibrio* NF, and to evaluate the effective usage of empirical antibiotics.

**Methods:**

One hundred and ninety‐four patients surgically confirmed with *Vibrio* NF were retrospectively reviewed, in which 180 (92.8%) patients were infected by *Vibrio vulnificus*. There were 179 patients (92.3%) with a monomicrobial infection and 15 (7.7%) with polymicrobial infections. Clinical characteristics, outcomes, laboratory data, and antibiotic usage were analyzed and compared between the two groups.

**Results:**

Twenty‐nine patients with *Vibrio* NF died (14.9%). Mortality was 15.6% (28/179) in the monomicrobial group and 6.7% (1/15) in the polymicrobial group. Among the 15 polymicrobial cases, 14 were caused by *Vibrio vulnificus* and 1 by *Vibrio parahaemolyticus*. Gout was more frequently observed in the polymicrobial group (4/15) than in the monomicrobial group (11/179). *Vibrio* isolates showed high susceptibility to third‐generation cephalosporins, fluoroquinolones, and tetracyclines. Effective antimicrobial therapy was associated with lower mortality (*p* = 0.002) and lower amputation rates (*p* = 0.004).

**Conclusions:**

Polymicrobial *Vibrio* NF was uncommon but was not clinically less severe than monomicrobial *Vibrio* NF in this 20‐year cohort. Patients with polymicrobial infection more frequently had gout, suggesting a potential clinical association that warrants further investigation. These findings indicate that suspected polymicrobial *Vibrio* NF should be treated with the same urgency as monomicrobial disease, with prompt surgery, intensive care, and empirical antimicrobial therapy covering both *Vibrio* species and relevant copathogens.

## 1. Introduction

Necrotizing fasciitis (NF) is a life‐threatening soft tissue infection with a high mortality rate of 10.3%–76% which need early diagnosis, emergent surgical intervention, intensive care, and effective antibiotic treatment [1–4]. NF is categorized as Type I (polymicrobial infection with aerobic and anaerobic bacteria), Type II (Group A *β*‐hemolytic *Streptococcus* spp. and *Staphylococcus aureus*), Type III (Gram‐negative pathogens), and Type IV (fungal infection) [1–8]. *Staphylococcus aureus* and group A *Streptococcus* spp. were the predominant pathogens causing NF in the United States and Europe; however, monomicrobial Gram‐negative pathogens, such as *Vibrio* spp., *Aeromonas* spp., *Escherichia coli*, and *Klebsiella* spp., were the most frequently isolated microorganisms in Asia [1–12]. *Vibrio* NF has been reported to present more clinical fulminant course and bullous skin progression than other pathogens, and *Vibrio* spp. were particularly concerning due to their rapid progression and high mortality rates of up to 18.6–42.9% [5–8].

*Vibrio* species naturally live in coastal areas, such as saltwater and brackish water, and can cause acute gastrointestinal diseases, necrotizing soft tissue infections (NSTIs), and primary sepsis in humans, which were typically associated with minor trauma and exposure to seawater or raw seafood [1, 3, 5–13]. Chiayi Chang Gung Memorial Hospital (CGMH) is a tertiary teaching hospital near Taiwan’s western coast where residents are often exposed to seawater, brackish water, and raw seafood, and *Vibrio vulnificus* (*V*. *vulnificus*) is the most frequent causative organism of monomicrobial NF [3, 9–13]. We set the *Vibrio* NSSTIs Treatment and Research (VTR) Group, including emergency medicine, orthopedics, infectious disease specialists, microbiologists, ICUs, plastic surgeons, and hyperbaric oxygen specialists, and have successfully decreased the mortality rate of *Vibrio* NF from 38% to 9.7% [5–7, 9, 12].

Although the clinical presentations of monomicrobial and polymicrobial NF were difficult to differentiate at initial presentation because of similar skin lesions, progressive erythematous changes, and pain out of proportion observed in the emergency department, we immediately performed emergent fasciotomy or limb amputation promptly based on clinical assessment, while definitive microbiological identification from blood and tissue cultures became available several days later [3, 5–7, 9, 12]. It was found that monomicrobial and polymicrobial NF exhibited significant differences in shock (hypotension at presentation, defined as systolic blood pressure < 90 mmHg), intensive care unit (ICU) admission, in‐hospital mortality, and 90‐day mortality [1, 2, 14]. Our previous studies revealed that those patients with polymicrobial *Aeromonas* NF had lower mortality rates than those with monomicrobial *Aeromonas* NF [10, 11]. Previous studies have primarily focused on monomicrobial *Vibrio* NF, general NSTI cohorts, or small case series involving *Vibrio* species; however, most studies did not specifically analyze polymicrobial *Vibrio* NF as a distinct subgroup [1–15]. Therefore, we focused on the exploratory and observational nature of the clinical findings between monomicrobial and polymicrobial *Vibrio* NF.

The purposes of this study were to compare the differences and the clinical outcomes between polymicrobial and monomicrobial *Vibrio* NF patients, to characterize polymicrobial *Vibrio* NF and compare its clinical features, microbiology, antimicrobial therapy, and outcomes with those of monomicrobial *Vibrio* NF, and to evaluate the effective usage of empirical antibiotic regimens.

## 2. Materials and Methods

### 2.1. Study Design

From January 2002 until December 2021, the VTR Group conducted a retrospective cohort study at CGMH‐Chiayi. A total of 261 patients were diagnosed with limb *Vibrio* soft tissue infections after arriving at the emergency room (ER). Of these, 64 patients with non‐NSTIs were excluded (Figure 1). Three patients were excluded due to a lack of admission data: one expired shortly after arrival at the ER, and two transferred to other hospitals before admission. A total of 194 patients with *Vibrio* NF of extremities were included in the study.

**FIGURE 1:**
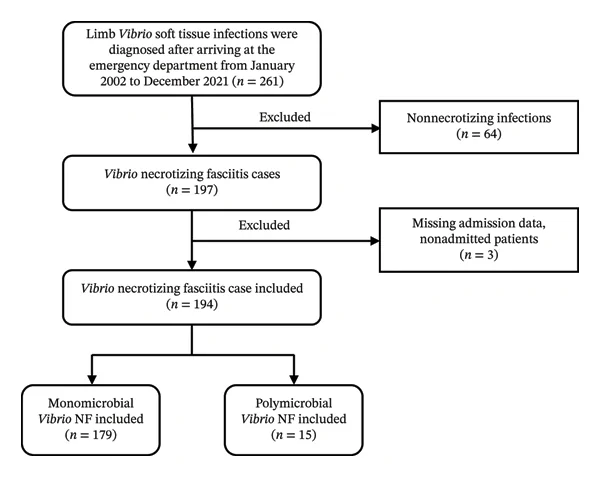
Flowchart of 194 patients with *Vibrio* necrotizing fasciitis.

### 2.2. Clinical Assessment

We have established the treatment strategy and protocol including emergency fasciotomy or amputation, initial empiric antibiotic therapy with a third‐generation cephalosporin with/without other regimens, and admission to the ICU for patients with fulminant clinical presentation wherein NF was diagnosed at ER. The study enrolled patients with *Vibrio* NF of limbs based on the following criteria: (1) The diagnosis of NF was confirmed by surgical findings, which included grayish necrotic skin, subcutaneous fat, and fascia, as well as a purulent discharge reminiscent of dishwasher odors [8, 15]; (2) histopathological examination of the tissue confirmed the diagnosis [16]; (3) *Vibrio* species were isolated from soft tissue lesions and blood collected immediately after the patient arrived in the ED or during surgery [8, 15]; and (4) limbs were infected by these bacteria.

Routinely, blood cultures were collected in the ER, and wound cultures were obtained during the first surgery. The patients were divided into monomicrobial and polymicrobial groups based on the infecting organism identified in their cultures. The monomicrobial NF was demonstrated by single *Vibrio* infection, and the polymicrobial NF was defined as *Vibrio* spp. combined with other bacterial isolates in patients. *Vibrio* spp. was considered the predominant organisms when they demonstrated heavy growth (3+ or 4+ in the semi‐quantitative grading system) and clearly exceeded the growth of other coisolated organisms in colony number and surface coverage on the agar plates. When other organisms were present only in light or moderate quantities (1+ or 2+ growth), *Vibrio* spp. were classified as the predominant pathogens in culture.

Based on antimicrobial susceptibility testing, effective antimicrobial usage is defined as supplying coverage to all infectious isolates, and combination therapy was defined as third‐generation cephalosporin with doxycycline or fluoroquinolones [17, 18]. The associations of effective antimicrobial therapy, combination therapy, and third‐generation cephalosporin plus doxycycline with mortality and amputation rates were analyzed. The patients’ characteristics, demographics, chronic illness history, clinical presentations with laboratory data at the time of admission to the ER, operation records, microorganisms, antibiotic regimens, usage, and outcomes were compared between the two groups.

### 2.3. Microbiology Laboratory Procedures

Primary culture isolates from wound and blood specimens were identified by Gram staining, serological agglutination, and conventional biochemical tests in the earlier study period. In the past decade, species identification was further confirmed using matrix‐assisted laser desorption/ionization time‐of‐flight mass spectrometry (MALDI‐TOF MS) with the Bruker Microflex LT/SH Biotyper system. *Vibrio* isolates were identified based on colony morphology on TCBS agar, Gram staining, serological agglutination with specific antisera, and biochemical characteristics. The isolates were Gram‐negative straight or slightly curved motile rods, oxidase‐ and catalase‐positive, sensitive to the vibriostatic agent O/129 (150 µg), and fermentative. Conventional biochemical tests included oxidase, H_2_S production, motility, indole, methyl red, Voges–Proskauer, and salt tolerance tests. Further confirmation was performed using the API 20E system (bio‐Mérieux Inc., Hazelwood, MO, USA) according to the manufacturer’s protocol. Antimicrobial susceptibility testing was performed using routine clinical laboratory methods according to the contemporaneous guidelines and breakpoint criteria of the Clinical and Laboratory Standards Institute throughout the study period.

### 2.4. Statistical Analysis

We used the Student’s *t*‐test and ANOVA for continuous variables and the Fisher exact test for categorical variables. The significance of the results was determined by *p* values < 0.05 with two‐tailed tests. Data were analyzed using IBM SPSS Statistics Version 20. Multivariable logistic regression analysis was performed to identify independent predictors of mortality between monomicrobial and polymicrobial NF. Unadjusted odds ratio analysis and multivariable logistic regression analysis were performed in a stepwise manner to determine the significance of effective antimicrobial therapy and clinical outcomes. Variables with *p* value < 0.05 in univariate analysis or with clinical relevance were included in the regression model. Adjusted odds ratios (aORs) with 95% confidence intervals (CIs) were reported.

## 3. Results

Twenty‐nine patients died with a mortality rate of 14.9% (29/194), and 18 patients had received amputation with an amputation rate of 9.3% (18/194). There were 179 patients (92.3%) with a monomicrobial infection, and 15 patients (7.7%) with a polymicrobial infection. All *Vibrio* isolates were susceptible to ceftazidime, ceftriaxone, levofloxacin, and tetracycline.

### 3.1. Characteristics of the Monomicrobial and Polymicrobial Groups

Patients were classified into monomicrobial and polymicrobial groups according to culture results, with the polymicrobial group defined by the isolation of *Vibrio* species together with additional microorganisms from blood or wound cultures. The two groups showed similar baseline characteristics, ICU requirements, limb involvement, and Laboratory Risk Indicator for Necrotizing Fasciitis (LRINEC) scores (Table 1). Culture findings demonstrated that NF was most commonly associated with *V. vulnificus*, which was identified in 179 patients (92.3%). Positive *Vibrio* cultures were identified from blood specimens only in 50 monomicrobial and 1 polymicrobial cases, from wound specimens only in 68 and 7 cases, respectively, and from both blood and wound specimens in 61 and 7 cases, respectively. Mortality was 15.6% (28/179) in the monomicrobial group and 6.7% (1/15) in the polymicrobial group. The single fatal polymicrobial case involved *V*. *vulnificus* with *Aeromonas hydrophila* and *Enterococcus faecalis.* The isolates of *Vibrio* species in the polymicrobial group were 14 of *V*. *vulnificus* and 1 of *V*. *parahaemolyticus*.

**TABLE 1:** Patient characteristics and demographic data for monomicrobial and polymicrobial *Vibrio* necrotizing fasciitis.

Variable	Monomicrobial (*n* = 179)	Polymicrobial (*n* = 15)	*p* value
Age (years)	65.9 ± 13.5	61.1 ± 9.7	0.188
Gender (male)	130 (72.6)	12 (80.0)	0.536
Involved lower limbs	92 (51.4)	9 (60.0)	0.791
Need ICU^a^ care	104 (58.1)	8 (53.3)	0.720
LRINEC^b^ score	3.0 ± 2.3	3.5 ± 3.2	0.457
Positive culture			
Blood	50	1	
Wound	68	7	
Blood and wound	61	7	
*Vibrio* species (dead)			
*Vibrio vulnificus*	165 (22)	14 (1)	
*Vibrio parahaemolyticus*	5 (1)	1 (0)	
*Vibrio cholerae* non‐O1	6 (4)	0	
*Vibrio fluvialis*	2 (1)	0	
*Vibrio mimicus*	1 (0)	0	
Chronic underlying diseases			
Chronic alcoholism	17 (9.5)	1 (6.7)	0.717
Chronic liver disease	64 (35.8)	4 (26.7)	0.479
Viral hepatitis	49 (27.4)	4 (26.7)	0.953
HBV	22 (12.3)	2 (13.3)	0.906
HCV	35 (19.6)	2 (13.3)	0.556
Liver cirrhosis	28 (15.6)	1 (13.3)	0.349
Chronic kidney disease	11 (6.1)	0 (0)	0.323
Cancer	10 (5.6)	1 (6.7)	0.862
Cerebrovascular disease	4 (2.2)	1 (6.7)	0.298
Cardiovascular disease	56 (31.3)	5 (33.3)	0.870
Diabetes mellitus	52 (29.1)	6 (40.0)	0.374
Gout	11 (6.1)	4 (26.7)	0.004^∗^

*Note:* Data were presented as the mean (standard deviation) or frequency (%).

^a^ICU, intensive care unit.

^b^LRINEC, Laboratory Risk Indicator for Necrotizing Fasciitis.

^∗^ *p* < 0.05.

The polymicrobial group had a higher incidence of lower limb infections than the monomicrobial group, but it was not statistically different. The polymicrobial group had a higher incidence of gout than the monomicrobial group (26.7% vs. 6.1%; *p* = 0.004), in which one patient from the monomicrobial group and one patient from the polymicrobial group died. Among the four patients in the polymicrobial group with a medical history of gout, three patients were coinfected with Gram‐positive pathogens and one patient was coinfected with Gram‐negative pathogens.

### 3.2. Comparison of Laboratory Test Results and Clinical Outcomes of the Two Groups

We compared initial disease severity, laboratory findings, treatment timing, and clinical outcomes between the monomicrobial and polymicrobial groups. No significant differences were observed in the interval from symptom onset to emergency department presentation, presenting clinical features, laboratory findings, time from diagnosis to first surgery, mortality, amputation, number of debridement operations, or length of hospital stay (Table 2). The mortality and amputation rates in the polymicrobial group were lower than those in the monomicrobial group, but this difference was not statistically significant. The number of operations, hemorrhagic bullae formation, skin necrosis, and hospital stays were similar in both groups (Figures 2 and 3).

**TABLE 2:** The clinical presentation, laboratory data, and outcomes between monomicrobial and polymicrobial *Vibrio* necrotizing fasciitis.

Variable	Monomicrobial (*n* = 179)	Polymicrobial (*n* = 15)	*p* value
The interval from symptom onset to emergency room presentation (days)	1.2 ± 1.1	1.2 ± 1.1	0.985
Clinical presentation			
Fever (body temperature > 38°C)	78 (43.6)	9 (60.0)	0.219
Tachycardia (heart rate > 100 beats/min)	112 (62.6)	11 (73.3)	0.406
Tachypnea (respiratory rate > 20 breaths/min)	70 (39.1)	5 (33.3)	0.659
Shock (systolic blood pressure < 90 mmHg)	84 (46.9)	4 (26.7)	0.130
Hemorrhagic bullae	76 (42.5)	5 (33.3)	0.491
Skin necrosis	60 (33.5)	2 (13.3)	0.107
Bacteremia	111 (62.0)	9 (60.0)	1.000
*Vibrio vulnificus* bacteremia	102 (57.0)	8 (53.3)	0.793
Laboratory test results			
Total WBC^a^ count			
Leukocytosis (> 12000/uL)	89 (49.7)	8 (53.3)	0.804
Leukopenia (< 4000/uL)	12 (6.7)	0 (0)	0.299
Leukocytosis or leukopenia	101 (56.4)	8 (53.3)	0.798
Differential count			
Band forms > 10%	53 (29.6)	4 (26.7)	0.810
Neutrophilia (> 7500/uL)	112 (62.6)	11 (73.3)	0.406
Lymphocytopenia (< 1000/uL)	115 (64.2)	9 (60.0)	0.742
Anemia (hemoglobin < 10 g/dL)	13 (7.3)	1 (6.7)	0.932
Thrombocytopenia (platelet count < 15 × 104 uL)	107 (59.8)	7 (46.7)	0.322
PT^b^ (seconds)	14.0 ± 5.2	12.7 ± 4.2	0.388
APTT^c^ (seconds)	34.2 ± 11.5	32.1 ± 5.4	0.528
Albumin (mg/dL)	2.5 ± 1.4	2.7 ± 1.3	0.635
Total bilirubin (mg/dL)	2.1 ± 2.9	1.3 ± 0.7	0.374
Creatine (μmol/L)	139.7 ± 98.9	132.5 ± 71.6	0.784
C‐reactive protein (mg/dL)	65.2 ± 81.6	82.2 ± 130.9	0.466
Time (hrs) from ER^d^ to the OR^e^	30.4 ± 64.8	34.8 ± 51.2	0.803
Mortality cases	28 (15.6)	1 (6.7)	0.703
Amputation cases	18 (10.1)	0 (0)	0.197
Number of debridement operations	3.0 ± 1.7	3.0 ± 1.8	0.900
Length of hospital stay (days)	32.4 ± 20.1	31.8 ± 15.9	0.918

*Note:* Data were presented as the mean (standard deviation) or frequency (%).

^a^WBC, white blood cell.

^b^PT, prothrombin time.

^c^APTT, activated partial thromboplastin time.

^d^ER, emergency room.

^e^OR, operation room.

**FIGURE 2:**
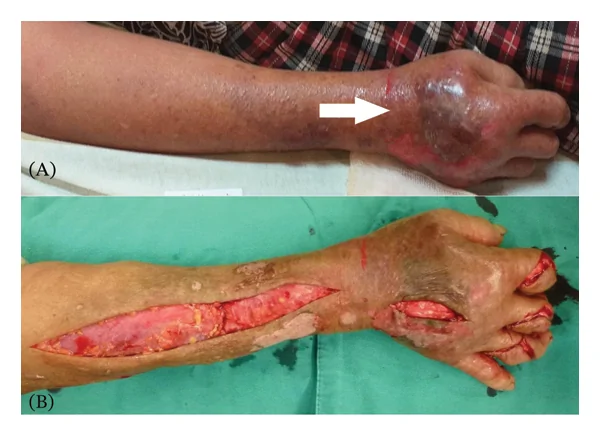
Clinical presentation of *Vibrio vulnificus* necrotizing fasciitis in an 80‐year‐old woman with chronic hepatitis B. (A) The right forearm and hand on arrival at the emergency department show extensive purpura and hemorrhagic bullae (white arrow). (B) Emergency fasciotomy was performed 4 h after surgical consultation. Blood and wound cultures yielded monomicrobial *Vibrio vulnificus* infection. Despite treatment, the patient died from progressive sepsis on hospital day 7.

**FIGURE 3:**
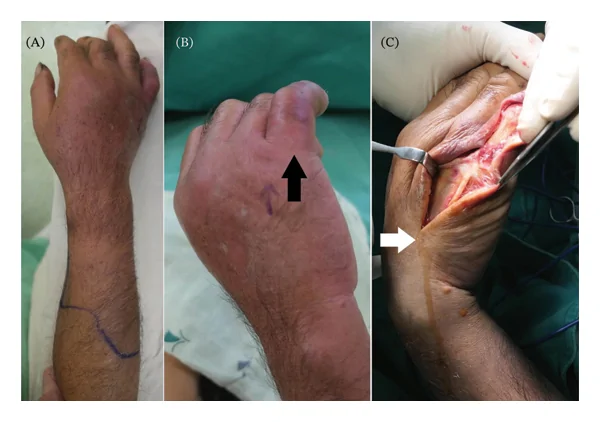
Clinical presentation of polymicrobial necrotizing fasciitis in a 67‐year‐old man with chronic hepatitis B presented with a 3‐day history of right‐hand pain and swelling. (A, B) Photographs obtained before surgery show purpura and hemorrhagic bullae over the right hand and fifth finger (black arrow). (C) Emergency fasciotomy revealed dishwater‐colored serous fluid in the wound (white arrow). Wound cultures grew *Vibrio vulnificus* and *Kocuria kristinae*. The patient underwent a second debridement 21 days after fasciotomy and was discharged on hospital day 32.

Multivariable logistic regression analysis was performed to adjust for potential confounding variables, such as age, liver disease, liver cirrhosis, shock, bacteremia, timing to operation, gout, and effective empirical therapy (Table 3). Shock (systolic blood pressure < 90 mmHg) was independently associated with increased mortality (aOR 3.82, 95% CI 1.75–8.36, *p* = 0.001), whereas effective empirical antimicrobial therapy was associated with lower mortality (aOR 0.34, 95% CI 0.13–0.88, *p* = 0.026). Gout was also an independent predictor of mortality (aOR 3.85, 95% CI 1.12–13.25, *p* = 0.033). Age and chronic liver disease were not significantly associated with mortality. Bacteremia, although common, was not associated with mortality and was excluded from the multivariable model. Polymicrobial infection was not independently associated with mortality.

**TABLE 3:** Multivariable logistic regression analysis for mortality between monomicrobial and polymicrobial *Vibrio* necrotizing fasciitis.

Variable	Adjusted odds ratio (aOR)	95% confidence interval	*p* value
Age (per year increase)	1.01	0.98–1.04	0.41
Shock	3.82	1.75–8.36	0.001^∗^
Gout	3.85	1.12–13.25	0.033^∗^
Chronic liver disease	0.91	0.33–2.55	0.86
Polymicrobial infection	0.58	0.07–4.60	0.60
Effective empirical antimicrobial therapy	0.34	0.13–0.88	0.026^∗^

^∗^Statistically significant (*p* < 0.05).

### 3.3. Microbiological Analysis of the Polymicrobial Group

Ten patients with polymicrobial *Vibrio* NF had one additional coisolated organism and five patients were coinfected with more than two pathogens. All 25 coinfective pathogens were isolated from wound specimens, of which 22 (88%) were aerobic bacteria. The most common isolates obtained from the polymicrobial group were *Staphylococcus* species (4, 16%), *Shewanella putrefaciens* (3, 12%), and *Enterococcus* spp. (3, 12%) (Table 4).

**TABLE 4:** Summary of identified infectious microorganisms in 15 patients with *Vibrio* polymicrobial NF.

Identified infectious microorganisms	Total no.	(%)
Gram‐positive aerobic pathogens	9	36
* Staphylococcus* spp.	4	16
MRSA^a^	2	8
* Staphylococcus epidermidis*	1	4
* Staphylococcus saprophyticus*	1	4
* Enterococcus* spp.	3	12
* Streptococcus dysgalactiae*	1	4
* Kocuria kristinae*	1	4
Gram‐negative aerobic pathogens	13	52
* Shewanella putrefaciens*	3	12
* Aeromonas* spp.	2	8
* Pseudomonas aeruginosa*	2	8
* Escherichia coli*	1	4
* Enterobacter cloacae*	1	4
* Klebsiella pneumoniae*	1	4
* Morganella morganii*	1	4
* Proteus vulgaris*	1	4
* Serratia marcescens*	1	4
Anaerobic pathogens	3	12
* Clostridium* spp.	1	4
* Finegoldia magna*	1	4
* Peptoniphilus harei*	1	4
Total	25	100

^a^MRSA: methicillin‐resistant *Staphylococcus aureus.*

### 3.4. Antibiotic Sensitivity and Empirical Antibiotic Selection Between Two Groups

Table 5 shows that susceptibility testing was reported for individual antimicrobial agents or antimicrobial classes with varying denominators according to the number of isolates tested. In both groups, *Vibrio* isolates demonstrated high susceptibility to third‐generation cephalosporins, fluoroquinolones, tetracyclines, carbapenems, aminoglycosides, and sulfonamides. Ampicillin susceptibility was significantly lower in the polymicrobial group than in the monomicrobial group (50.0% vs. 90.7%, *p* = 0.016).

**TABLE 5:** Antimicrobial susceptibility testing and the initial empiric antibiotic usage at ER between monomicrobial and polymicrobial *Vibrio* necrotizing fasciitis.

Variable	Monomicrobial (*n* = 179)	Polymicrobial (*n* = 15)	*p* value
Coinfection with Gram‐positive bacteria	0	9	
Coinfection with Gram‐negative bacteria	0	13	
Antibiotic sensitivity of *Vibrio* spp.^∗^			
Ampicillin	49/54 (90.7)	2/4 (50)	0.016^∗∗^
Aminoglycosides	77/79 (97.5)	7/7 (100)	0.670
Third‐generation cephalosporins	179/179 (100)	15/15 (100)	1.000
Fluoroquinolones	171/173 (98.8)	15/15 (100)	0.675
Tetracyclines	102/103 (99.0)	9/9 (100)	0.767
Carbapenems	79/79 (100)	7/7 (100)	1.000
Sulfa drugs	59/61 (96.7)	7/7 (100)	0.627
Effective empirical antimicrobial usage	153 (85.5)	11 (73.3)	0.212
Monotherapy	88 (49.2)	8 (53.3)	0.756
Third‐generation cephalosporins	87 (48.6)	8 (53.3)	0.725
Fluoroquinolones	1 (0.6)	0 (0.0)	0.772
Combination therapy	65 (36.3)	3 (20.0)	0.203
Third‐generation cephalosporins + doxycycline	61 (34.1)	3 (20.0)	0.265
Third‐generation cephalosporins + fluoroquinolones	4 (2.2)	0 (0.0)	0.559
Ineffective empirical antimicrobial usage	26 (14.5)	4 (26.7)	0.212

*Note:* Data were presented as frequency (%).

^∗^Data not all available.

^∗∗^ *p* < 0.05.

Effective empirical antimicrobial therapy was administered in 85.5% of monomicrobial cases and 73.3% of polymicrobial cases, with no significant difference between the groups. Monotherapy with third‐generation cephalosporins was the most commonly used regimen, whereas combination therapy mainly consisted of third‐generation cephalosporins plus doxycycline. Eleven patients (73.3%) in the polymicrobial group using our empirical antimicrobial regimens were found effective for coverage of both *Vibrio* spp. and non‐*Vibrio* organisms.

Those patients who received effective antimicrobial therapy, combination therapy, and third‐generation cephalosporin plus doxycycline were found to have a significant association with lower mortality. Those patients who were treated with effective antimicrobial therapy had significantly associated with lower incidence of amputation (Table 6). Unadjusted odds ratio analysis and multivariable logistic regression analysis demonstrated that effective antimicrobial therapy was associated with significantly lower mortality. Similarly, combination therapy and third‐generation cephalosporin plus doxycycline therapy were associated with reduced odds of death. Effective antimicrobial therapy was also associated with a lower risk of amputation (Table 7).

**TABLE 6:** Clinical outcome and initial empiric antibiotic usage in *Vibrio* NF patients.

Variable	Effective antimicrobial usage		*p* value	Combination therapy		*p* value	Third‐generation cephalosporin + DOX		*p* value
	Yes (*n* = 164)	No (*n* = 30)		Yes (*n* = 68)	No (*n* = 126)		Yes (*n* = 64)	No (*n* = 130)	
Mortality	19 (11.6)	10 (33.3)	0.002^∗^	3 (4.4)	26 (20.6)	0.002^∗^	2 (3.1)	27 (20.8)	0.001^∗^
Amputation	11 (6.7)	7 (23.3)	0.004^∗^	4 (5.9)	14 (11.1)	0.231	3 (4.7)	15 (11.5)	0.122

*Note:* DOX: doxycycline. Data were presented as frequency (%).

^∗^ *p* < 0.05.

**TABLE 7:** Association between empirical antimicrobial therapy and clinical outcomes by multivariable logistic regression analysis.

Outcome	Variable (total 194)	Univariate OR (95% CI)	*p* value	Multivariable aOR (95% CI)	*p* value
Mortality	Effective antimicrobial usage	0.26 (0.11–0.64)	0.002^∗^	0.34 (0.13–0.88)	0.026^∗^
	Combination therapy	0.18 (0.05–0.61)	0.002^∗^	0.22 (0.06–0.78)	0.019^∗^
	Third‐generation cephalosporin + doxycycline	0.12 (0.03–0.54)	0.001^∗^	0.21 (0.05–0.89)	0.034^∗^

Amputation	Effective antimicrobial usage	0.24 (0.08–0.67)	0.004^∗^	0.31 (0.10–0.98)	0.046^∗^
	Combination therapy	0.50 (0.16–1.59)	0.231	0.58 (0.18–1.88)	0.37
	Third‐generation cephalosporin + doxycycline	0.38 (0.11–1.35)	0.122	0.44 (0.12–1.62)	0.21

^∗^Statistically significant (*p* < 0.05).

## 4. Discussion

The principal findings of this study were that patients with polymicrobial *Vibrio* NF had clinical characteristics, laboratory findings, and outcomes similar to those of patients with monomicrobial *Vibrio* NF. Among the 194 patients with *Vibrio* NF, 15 (7.7%) had polymicrobial infections, and only one patient in this group died. *Vibrio vulnificus* was the predominant *Vibrio* species identified in both groups. In addition, gout was more frequently observed among patients with polymicrobial infection. Several cohort studies have reported similar or even higher mortality among monomicrobial infections compared with polymicrobial disease, reflecting differences in pathogen virulence and host factors [1–3, 12, 14, 16, 19]. However, those studies specifically focusing on polymicrobial *Vibrio* NF remain limited [20–25]. Therefore, we compared mortality‐associated factors, clinical presentations, laboratory findings, and antimicrobial therapy between patients with monomicrobial and polymicrobial *Vibrio* NF. To our knowledge, this study represents one of the largest clinically focused cohorts examining polymicrobial *Vibrio* NF and provides additional information regarding its clinical characteristics and outcomes.

Polymicrobial *Vibrio* NF was uncommon in our cohort, accounting for 15 of 194 cases (7.7%). These polymicrobial cases frequently involved additional Gram‐positive and Gram‐negative organisms, while the clinical characteristics, laboratory findings, and outcomes were broadly comparable to those of monomicrobial *Vibrio* NF. These findings indicate that polymicrobial *Vibrio* NF represents an uncommon but clinically important presentation that requires prompt recognition and management. Because the present study was based on clinical and culture data, the biological mechanisms underlying the coexistence of multiple microorganisms could not be determined.

By performing early fasciotomy, empiric third‐generation cephalosporin antibiotic therapy and ICU care, the mortality rate in the *Vibrio* polymicrobial group was lower than those in the monomicrobial group (6.7% vs.15.6%); nevertheless, the two groups did not show statistically significant differences in mortality rate, chronic underlying diseases, and lab data. Although crude mortality was numerically lower in the polymicrobial group than in the monomicrobial group, this difference should be interpreted with caution because the polymicrobial group included only 15 patients and one mortality event. The present study was underpowered to detect clinically meaningful differences in mortality between groups. Therefore, these findings should not be interpreted as evidence that polymicrobial *Vibrio* NF has a more favorable prognosis or is clinically less severe than monomicrobial infection. Instead, our results suggest that polymicrobial *Vibrio* NF should be managed with the same degree of clinical urgency as monomicrobial disease until larger studies provide more definitive evidence.

In the present cohort, *Vibrio* isolates demonstrated high susceptibility to aminoglycosides, third‐generation cephalosporins, fluoroquinolones, tetracyclines, carbapenems, and sulfonamides. Effective empirical antimicrobial therapy was administered in 153 (85.5%) patients with monomicrobial *Vibrio* NF and 11 (73.3%) patients with polymicrobial *Vibrio* NF. Because polymicrobial infections commonly involved additional Gram‐positive and Gram‐negative organisms, adequate empirical antimicrobial coverage should include both *Vibrio* species and potential copathogens. In our cohort, the empirical antimicrobial protocol provided appropriate coverage for both *Vibrio* and most coisolated organisms in the majority of polymicrobial cases. Although the frequency of initially inadequate empirical antimicrobial therapy was higher in the polymicrobial group than in the monomicrobial group (26.7% vs. 14.5%), antimicrobial regimens were subsequently adjusted according to culture and susceptibility results under the guidance of infectious disease specialists. Consequently, no significant differences in antimicrobial usage or mortality were observed between the two groups in univariable analyses. The susceptibility patterns observed in our cohort were generally consistent with previous reports from Taiwan, where *Vibrio* species remain highly susceptible to third‐generation cephalosporins, aminoglycosides, fluoroquinolones, and tetracyclines [5–11, 22, 26]. In contrast, studies from the Maryland Coastal Bays in the United States have reported higher resistance rates to third‐generation cephalosporins and carbapenems among some *Vibrio* isolates [27]. These findings highlight potential geographic differences in antimicrobial susceptibility and support the importance of regional surveillance to guide empirical antimicrobial therapy.

In multivariable logistic regression analysis, shock was independently associated with increased mortality (aOR 3.82, 95% CI 1.75–8.36, *p* = 0.001), whereas effective empirical antimicrobial therapy, combination therapy, and third‐generation cephalosporin plus doxycycline therapy were associated with lower mortality. The application of the effective antimicrobial agents under the supervision of infectious disease specialists can improve the outcomes of patients with *Vibrio* NF, especially when NF patients presented shock at ER; however, because treatment allocation was not randomized, these associations should not be interpreted as evidence of causality.

Third‐generation cephalosporins, particularly ceftriaxone, were the most commonly prescribed empirical antimicrobial agents in our institution, and our previous studies demonstrated that ceftriaxone‐containing regimens were associated with favorable outcomes in patients with NF presenting with bloodstream infection and hemorrhagic bullae [7–9, 13, 26]. In the present cohort, third‐generation cephalosporins and fluoroquinolones showed excellent in vitro activity against *Vibrio* isolates and were considered appropriate empirical agents for suspected *Vibrio* NF. However, because polymicrobial infections may involve additional Gram‐positive organisms, Gram‐negative organisms, and anaerobes, antimicrobial therapy was adjusted according to culture and susceptibility results when necessary [28–31]. The principal clinical implication of the present study is that empirical antimicrobial therapy for suspected polymicrobial *Vibrio* NF should provide coverage for both *Vibrio* species and clinically relevant copathogens until culture and susceptibility results become available. However, no conclusions can be drawn regarding the independent effect of infectious disease specialist involvement because this factor was not specifically evaluated, and these findings should be interpreted cautiously because of the retrospective observational design of the study. In addition to antimicrobial therapy, early surgical intervention, intensive care management, and multidisciplinary care likely contributed substantially to patient outcomes.

One notable finding of this study was that a history of gout was more frequently observed among patients with polymicrobial *Vibrio* NF. Several mechanisms have been proposed to explain the potential association between gout and susceptibility to polymicrobial infection, including chronic inflammation, metabolic comorbidities, and skin barrier disruption. Some studies have also reported associations between gout and Gram‐positive skin and soft tissue infections, including Group A *Streptococcus* and *Staphylococcus aureus* [32–36]. Although a history of gout was more frequently observed in the patients with polymicrobial *Vibrio* NF, this finding was based on only four cases and should be interpreted cautiously, and these hypotheses remain speculative because the present study did not evaluate gout severity, tophus formation, skin lesions, serum uric acid levels, medication use, or other relevant clinical factors. Clinically, patients with gout, particularly those with skin barrier disruption or soft tissue vulnerability, may require careful evaluation following wound exposure, seawater exposure, or seafood‐related injury in coastal regions.

There were several limitations to the present study. First, this was a retrospective observational study conducted at a single institution and was therefore subject to potential selection bias and unmeasured confounding. In addition, the relatively small number of polymicrobial *Vibrio* NF cases (*n* = 15) limited the statistical power and precision of between‐group comparisons. Therefore, nonsignificant findings should be interpreted cautiously, as they may reflect insufficient power to detect clinically meaningful differences rather than true equivalence between the monomicrobial and polymicrobial groups. Second, the study did not include detailed molecular, genetic, or functional investigations of the bacterial isolates, including minimum inhibitory concentration distributions, resistance mechanisms, genotyping, or polymerase chain reaction‐based analyses. Therefore, the contribution of specific *Vibrio* virulence mechanisms and potential pathogen–pathogen interactions in polymicrobial infections could not be assessed, and no mechanistic conclusions can be drawn from the present clinical and culture‐based data. Further studies incorporating molecular and genomic approaches are warranted to better characterize the microbiological characteristics and potential interactions of polymicrobial *Vibrio* infections. Third, only 29 mortality events occurred overall, including one in the polymicrobial group. The limited number of outcome events may have reduced the stability and precision of the multivariable logistic regression estimates and increased the risk of model overfitting. Although variables were selected based on clinical relevance and univariable findings, the limited event‐to‐variable ratio warrants cautious interpretation of the multivariable results. Therefore, the results of the multivariable models should be interpreted cautiously and require validation in larger cohorts.

## 5. Conclusions

Polymicrobial *Vibrio* NF was uncommon but was not clinically less severe than monomicrobial *Vibrio* NF in this 20‐year cohort. Patients with polymicrobial infection more frequently had gout, suggesting a potential clinical association that warrants further investigation. These findings indicate that suspected polymicrobial *Vibrio* NF should be treated with the same urgency as monomicrobial disease, with prompt surgery, intensive care, and empirical antimicrobial therapy covering both *Vibrio* species and relevant copathogens.

## Author Contributions

Jiun‐Liang Chen: conceptualization. Hung‐Yen Chen: conception and design of the study and drafting the article. Chun‐Hao Chen: acquisition of data. Hung‐Yen Chen: analysis and interpretation of data. Chien‐Hui Hung & Shu‐Fang Kuo: design and coordination. Hsing‐Jung Li: revising it critically for important intellectual content. Cheng‐Ting Hsiao: final approval of the version to be submitted. Tsung‐Yu Huang: writing review and editing.

## Funding

This work was supported by grants from the Chang Gung Memorial Hospital in Taiwan (Grant numbers CMRPG6P0031, CMRPG6N0061, CMRPG6L0071, and CMRPG6H0641 to Tsung‐Yu Huang).

## Disclosure

All authors read and approved the final manuscript for publication.

## Ethics Statement

The study was conducted according to the guidelines of the Declaration of Helsinki and approved by the Institutional Review Board of Chang Gung Medical Foundation (Numbers: 202301943B1, 202300428B1, 202001656B0, and 201801530B1B0).

## Conflicts of Interest

The authors declare no conflicts of interest.

## Data Availability

The datasets generated during and/or analyzed during the current study are available from the corresponding author on reasonable request.
